# Effect of Unbleached Rice Straw Cellulose Nanofibers on the Properties of Polysulfone Membranes

**DOI:** 10.3390/polym11060938

**Published:** 2019-05-29

**Authors:** Mohammad Hassan, Ragab E. Abou Zeid, Wafaa S. Abou-Elseoud, Enas Hassan, Linn Berglund, Kristiina Oksman

**Affiliations:** 1 Cellulose and Paper Department & Centre of Excellence for Advanced Sciences, National Research Centre, 33 El-Behouth street, Dokki, Giza 12622, Egypt; r_abouzeid2002@yahoo.com (R.E.A.Z.); ws.abouelseoud@nrc.sci.eg (W.S.A.-E.); ea.elgarhy@nrc.sci.eg (E.H.); 2 Egypt Nanotechnology Centre, Cairo University, El-Shiekh Zayed, 6th October City 12588, Egypt; 3 Department of Engineering Sciences and Mathematics, Luleå University of Technology, SE 97187 Luleå, Sweden; linn.berglund@ltu.se; 4 Department of Mechanical and Industrial Engineering, University of Toronto, 5 Kings College Road, Toronto, ON M5S 3G8, Canada

**Keywords:** rice straw, cellulose nanofibers, unbleached pulp, polysulfone, membrane

## Abstract

In addition to their lower cost and more environmentally friendly nature, cellulose nanofibers isolated from unbleached pulps offer different surface properties and functionality than those isolated from bleached pulps. At the same time, nanofibers isolated from unbleached pulps keep interesting properties such as hydrophilicity and mechanical strength, close to those isolated from bleached pulps. In the current work, rice straw nanofibers (RSNF) isolated from unbleached neutral sulfite pulp (lignin content 14%) were used with polysulfone (PSF) polymer to make membrane via phase inversion. The effect of RSNF on microstructure, porosity, hydrophilicity, mechanical properties, water flux, and fouling of PSF membranes was studied. In addition, the prepared membranes were tested to remove lime nanoparticles, an example of medium-size nanoparticles. The results showed that using RSNF at loadings from 0.5 to 2 wt.% can significantly increase hydrophilicity, porosity, water flux, and antifouling properties of PSF. RSNF also brought about an increase in rejection of lime nanoparticles (up to 98% rejection) from their aqueous suspension, and at the same time, with increasing flux across the membranes. Tensile strength of the membranes improved by ~29% with addition of RSNF and the maximum improvement was obtained on using 0.5% of RSNF, while Young’s modulus improved by ~40% at the same RSNF loading. As compared to previous published results on using cellulose nanofibers isolated from bleached pulps, the obtained results in the current work showed potential application of nanofibers isolated from unbleached pulps for improving important properties of PSF membranes, such as hydrophilicity, water flux, rejection, and antifouling properties.

## 1. Introduction

Polysulfone (PSF) is one of the attractive polymers due to its good mechanical properties, thermal stability, chemical resistance, transparency, and flexibility [1]. PSF is used in different kinds of membranes such as anion-exchange membranes [2]; hemodialyzer membranes [3]; gas separation membranes [4]; pressure-driven membranes, i.e., ultra- and nanofiltration membranes [5]; osmotically-driven membranes for desalination and ions removal [6]; proton-exchange membranes fuel cells [7]; membranes for use in artificial organs [8]; and membranes used in dehydration of solvents by pervaporation [9].

To obtain membrane with sufficient porosity, the phase inversion technique, which depends on precipitation of PSF from its solution in a nonsolvent, is usually practiced. Nevertheless, the high hydrophobic nature of PSF leads to fouling problems when used in membranes [1]. To overcome this shortcoming, PSF-based polymers have been developed such as polyether sulfone and sulfonated PSF. This of course adds to the cost of the produced membranes. Some inorganic additives have been used to improve the hydrophilicity and performance of PSF membranes such as Al_2_O_3_ [10], TiO_2_ [11], and silica [12,13]. Another route to overcome the hydrophobicity of PSF and to produce high-flux membranes is to blend it with other relatively higher hydrophilic polymers. Compatibility between PSF and polymers used and their solubility are important factors which limit the number of candidates for that purpose. Different synthetic polymers such as polyvinylpyrrolidone [14], polyethylene glycol [15], and polyaniline-polyvinylpyrrolidone [16] have been used with PSF to improve the aforementioned shortcomings. 

Cellulose, as a natural polymer, either in form of microfibers and nanocellulose, e.g., cellulose nanofibers and cellulose nanocrystals, have been studied to improve the hydrophilicity and water flux properties of PSF since they could be dispersed in the same solvents of PSF, such as dimethyl acetamide. The presence of S=O polar groups in polysulfones helps in forming hydrogen bonding with hydroxyl groups of cellulose. For example, cellulose nanocrystals have been used to improve hydrophilicity, mechanical properties, and performance of polysulfone [17,18,19,20,21,22,23] and polyether sulfone [24]. Regarding use of cellulose nanofibers (CNF), CNF isolated from bleached cellulose fibers have been used to improve mechanical and hydrophilicity of PSF [23], PSF/sulfonated PSF membrane [25], and polyether sulfone [26]. Also, methacryloxypropyltrimethoxy silane-modified CNF have been used to improve mechanical properties and performance of PSF membranes [27]. 

In addition to cellulose, the use of lignin, which produced as a byproduct from pulping processes, is another attractive route for improving hydrophilicity of polysulfones due to the presence of polar groups in the lignin structure in addition to the aromatic skeleton, which helps in achieving good compatibility between lignin and polysulfones [28,29].

Motivated by the desirable effect of both lignin and cellulose on properties of polysulfones, Ding et al. [30] studied the use of what they called “lignin/cellulose nanofibers” complex prepared by sulfuric acid hydrolysis of unbleached pulp followed by submerging the neutralized treated pulp in dimethylacetamide and high pressure homogenization, for improving hydrophilicity and mechanical properties of polyethersulfone; the ratio of lignin to cellulose nanofibers was up to 1.2 wt.%. 

Furthermore, there is a recent interest in isolation of cellulose nanofibers from unbleached pulp with high lignin content using ultrafine grinding, where the nanofibers are isolated only by the action of the high shear force during grinding [31]. In addition to saving chemicals used in bleaching in case of using unbleached pulp, CNF with lignin at the surface could show good compatibility with polymers that do not have sufficient compatibility with nanofibers obtained from bleached pulp. 

For the best of our knowledge, all previous work on using CNF with PSF for making membranes was mainly focused on using CNF isolated from bleached pulps. In the current work, the use of cellulose nanofibers isolated from unbleached rice straw neutral sulfite pulp (containing ~14% lignin) by ultrafine grinding for improving hydrophilicity, porosity, and ultrafiltration performance of PSF membrane was investigated. In addition, the effect of the isolated nanofibers on mechanical properties was investigated. 

## 2. Experimental

### 2.1. Materials

Rice straw obtained from a local farm in Qalubiyah, Egypt was washed with water to remove the dust and allowed to air dry. Sodium sulfite and sodium carbonate used for pulping were reagent grade chemicals and used as received. Dimethylacetamide (DMAc) and polysulfone with average M_w_ ~35,000 and, average M_n_ ~16,000 were used as received from Sigmaaldrich (St. Louis, MO, USA). Lyophilised bovine serum albumin (pH 7) was purchased from Biowest Company (Biowest, Naillé, France) and used as received.

### 2.2. Preparation of Rice Straw Pulp

Rice straw neutral sulfite pulp was prepared by pulping the straw using 10% sodium sulfite and 2% sodium carbonate (based on weight of rice straw) solutions at 160 °C for 2 h; the liquor ratio was 1:10. The produced pulp was thoroughly washed with water, defibrillated in a Valley beater (Valley Iron Works, Appleton, Wisconsin, USA) to a 25°SR degree of freeness, dewatered, and allowed to air dry. Chemical composition of the prepared pulp: 16.63% ash content, 14.15% Klason lignin, 3.24% acid insoluble lignin, 54.12% α-cellulose, 14.34% pentosans, and degree of polymerization: 903 [31].

### 2.3. Xylanases Pretreatment of Unbleached Rice Straw Pulps

Neutral sulfite unbleached pulp was pretreated with xylanases in citrate buffer (pH = 5.3) for 4 h at 50 °C as previously described [31]. The concentration of xylanase used was 0.04 g/g of pulp. Chemical composition of the pretreated pulp was 16.46% ash content, 13.18% Klason lignin, 2.31% acid insoluble lignin, 58.4% α-cellulose, 10.79% pentosans, and degree of polymerization: 1097 [31].

### 2.4. Isolation of Cellulose Nanofibers from Xylanase-Treated Unbleached Pulp

Isolation of cellulose nanofibers from unbleached pulp was carried out similar to the previously published protocol [31]. In brief, the unbleached pulp was first disintegrated using a shear mixer (Silverson L4RT, Silverson Machines Ltd., Chesham, UK) using pulp suspension of 2 wt.% consistency. The pulp was then fibrillated using high-shear ultrafine friction grinder, or a so-called Supermasscolloider (MKCA6-2, Masuko Sangyo, Kawaguchi, Japan).The gap between the disks was gradually adjusted to −90 µm and the pulp was run through the grinder for approximately 140 min. 

### 2.5. Preparation of PSF/RSNF Membrane

PSF solution (18 wt.%) was prepared by dissolving in DMAc. Water in the RSNF suspension was first removed by vacuum filtration then acetone was passed once through the filtered CNF, and finally DMAc was passed twice. The DMAc-wetted RSNF were kept in a closed container in fridge at 8 °C till use. The DMAc-wetted RSNF were added to PSF solution at ratios from 0.5 to 2 wt.% of dry RSNF to PSF; the mixture was homogenized by magnetic stirring for 30 min. The viscosity of the PSF/RSNF mixture was measured using a tuning-fork vibration viscometer (Vibro Viscometer SV-10, A&D Company Limited, Tokyo, Japan). The films were prepared by phase inversion in distilled water, washed thoroughly with distilled water, and left to dry in air.

### 2.6. Characterization of PSF/RSNF Membrane

Tensile testing was carried out on 1-cm-wide films using a Lloyd instrument (Lloyd Instruments, West Sussex, UK) with a 100-N load cell; a cross-head speed of 2 mm/min was used and the gauge length was 20 mm. Five replicates of each sample were used and the results averaged. The water contact angle of films was measured using an Attension theta lite measuring system (Biolin Scientific AB, Gothenburg, Sweden) and calculated with the drop shape analysis OneAttension Version 2.7 (r5433), using a sessile drop technique. A 4 μL water drop was placed onto the films at four separate places for calculating the average contact angles. Microscopic features of films were investigated using a FEI Quanta 200 scanning electron microscope (FEI Company, Eindhoven, The Netherlands) at an acceleration voltage of 20 kV.

### 2.7. Preparation and Characterization of Lime Nanoparticles Suspension

Lime nanoparticles were prepared according to the previously published method [32]. In brief, to 100 mL containing 0.3 mol/L of calcium chloride, 0.6 mol/L of sodium hydroxide was added dropwise (≈ 4 mL/min) at 90°C. The precipitated calcium hydroxide nanoparticles were centrifuged and washed with previously boiled distilled water. The suspension containing purified calcium hydroxide nanoparticles was flushed with nitrogen gas and kept in closed bottle until use. Transmission electron microscopy (TEM) was carried out using high-resolution transmission electron microscopy (HR-TEM) (JEM-2100 transmission electron microscope, JEOL, Tokyo, Japan). Energy-dispersive X-ray microanalysis was carried out using JEOL JXA 8040 A electron probe microanalyzer (JEOL, Tokyo, Japan).

### 2.8. Evaluation of Membranes Properties

#### 2.8.1. Porosity

The porosity (ε) of membranes was determined from water absorption of the different membranes. The membranes were submerged in distilled water for 18 h then weighed after wiping excess water. Then, the wet membranes were dried at 105 °C until no change in weight. Porosity was calculated according to the following equation [33].

Porosity (ε) = [(m_1_ − m_2_)/ρ.A.L]*100 (1)

where m_1_ and m_2_ are the weight of the wet and dry films, respectively; ρ is the water density (g/cm^3^); A is the effective area of the films (cm^2^); and L is the film thickness (cm). 

#### 2.8.2. Pure Water Flux and Fouling

The water flux of the membranes was measured using a dead-end stirred cell (Sterlitech HP4750, Sterlitech, Kent, WA, USA). Prior to the measurements, discs with a diameter of ~5 cm were cut out from the membranes and soaked in water for one hour to ensure equilibration of the membrane. The conditioned membranes were placed in the dead-end cell on a stainless steel porous support disc and water was passed through the membranes at 25 °C at a differential pressure of 30 Mpa, maintained using pressure water pump. The quantity of water that passed through the membrane for a defined time interval was weighed accurately and the flux was calculated (L/h/m^2^/MPa) for the active filtration area (14.6 cm^2^). To avoid reduction of water flux as a result of membrane compaction, back pressure was applied every 10 min.

To test fouling of the membranes, bovine serum albumin solution (1 g/L) was passed through the membrane for 10 min under pressure of 30 MPa and flux was calculated. Then, the membranes were washed briefly with distilled water and pure water was passed through the membranes under the same pressure and protein solution was passed again; the cycle was repeated for one hour.

#### 2.8.3. Removing Lime Nanoparticles

To test the efficiency of the prepared membranes in removing lime, suspensions containing 1 g/L was passed through the membrane under the same conditions mentioned above for testing pure water flux. The concentration of the nanoparticles in the filtrate was measured from following the turbidity using UV–Visible spectrometer (Shimadzu, Tokyo, Japan) at wavelength of 600 nm. Turbidity (T) was calculated from measuring absorbance at 500 nm: Turbidity = (A * 2.302)/L, where A is the absorbance and l is the path length (0.01 m). The rejection of the prepared membranes to remove the nanoparticles was calculated using the following formula.

Rejection (%) = [(Control T_500_ − sample T_500_)/Control T_500_] × 100 (2)

## 3. Results and Discussion

**Figure 1:**
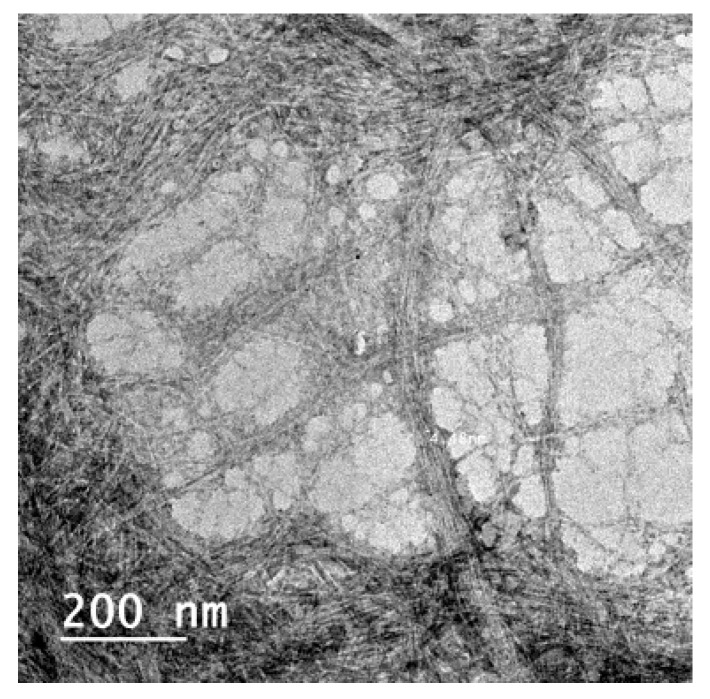
TEM image of cellulose nanofibers isolated from xylanase-treated rice straw unbleached neutral sulfite pulp.

In a previous publication, it has been shown that RSNF with high lignin content (~14 wt.%) could be isolated from rice straw xylanase-treated unbleached sulfite pulp with a width of ~14 ± 7 nm, as seen in Figure 1 [31]. Water in the isolated RSNF could be easily exchanged by filtration and simple washing using different non-aqueous aprotic solvent [34].

### 3.1. Effect of RSNF on Viscosity of PSF Solution

The effect of the addition of RSNF on viscosity of the PSF solution was studied as an indication of good dispersion of RSNF in the solution; the results are presented in Table 1. As shown in the figure, the viscosity of PSF solution increased with the addition of RSNF, in spite of the small concentrations of RSNF added (2% max.). The increase in viscosity ranged from 23% to 128% at the different RSNF loadings with respect to neat PSF solution.

### 3.2. Effect of RSNF on Microscopic Structure of PSF Membranes

**Figure 2:**
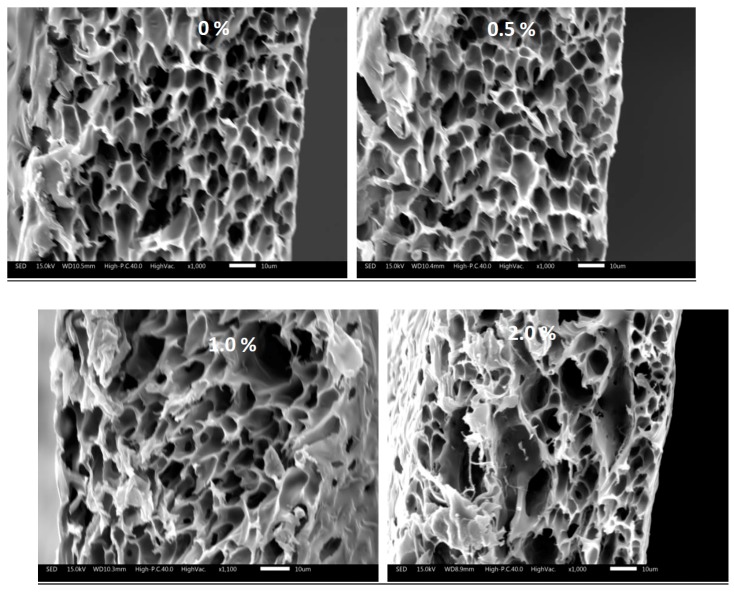
SEM of cross-section of polysulfone membranes with different RSNF loadings.

**Figure 3:**
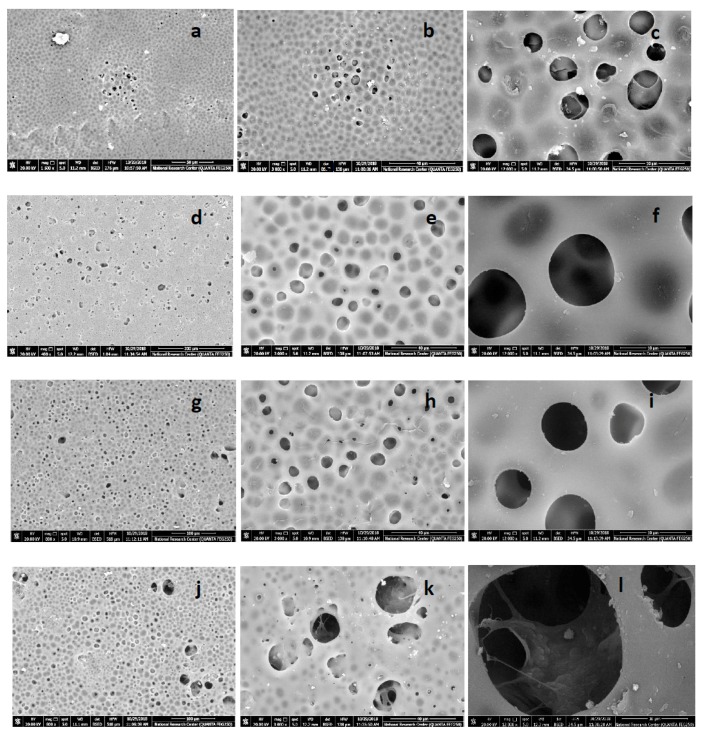
SEM image of surfaces of PSF (**a**–**c**), PSF/0.5% RSNF (**d**–**f**), PSF/1% RSNF (**g**–**i**), and PSF/2% RSNF (**j**–**l**) membranes at 800, 3000, and 12000x magnification, respectively (scale bar is 50, 40, and 10 µm, respectively).

Microscopic structure of polymeric membranes is very important since it determines both mechanical and filtration properties. The effect of RSNF on the microscopic structure of PSF membranes formed by phase inversion was studied by SEM; images of both surface and cross-section are shown in Figure 2 and Figure 3.

Regarding the cross-section, a slight change in the size of voids started to occur at RSNF loading of 1%, while at 2%, formation of larger and elongated slender-like voids was observed. On the other hand, the microscopic structure of PSF film’ surfaces clearly affected by the addition of RSNF. While neat PSF film had a few and very tiny pores at the surface, adding RSNF resulted in more and wider pores, especially at RSNF loading 1–2%. At higher magnification, SEM images of the surface of membrane sample containing 2% RSNF showed that although wide pores formed at the surface, the inner pores were narrower and with mesh-like structure.

Table 2 shows the average diameter of the pores at the surface of the different PSF samples seen from the images. Increasing the loading of RSNF resulted in larger pore diameter at the surface. Similar trend was observed in membranes prepared from cellulose nanofibers isolated from bleached pulp with polysulfone/sulfonated polysulfone mixture [24]. These changes in the microscopic features due to addition of cellulose nanofibers were interpreted by the accelerated phase inversion process by the hydrophilic cellulose nanofibers, which results in formation of more pores and better pores connectivity across the membrane. In addition, due to the large aspect ratio of cellulose nanofibers, they are easily aggregated during phase inversion and resulted in formation of pore defects in the membranes [18,25]. The same findings were also reported when methacryloxypropyltrimethoxy silane-modified cellulose nanofibers were used with PSF [27].

### 3.3. Effect of RSNF on Mechanical Properties of PSF Films

The effect of RSNF on tensile strength properties of PSF was studied and results are listed in Table 3. As the results show, adding RSNF resulted in a moderate increase in maximum tensile strength (~29%) at 0.5% RSNF loading followed by a decrease at higher RSNF loading, but still as high as that of neat PSF. This could be attributed to the more pores formed at the surface and pore defects across the thickness. The high standard deviations values at 2% RSNF loading can be attributed to the high porosity of the films, and thus failure of films was non-reproducible from one sample to another. On the other hand, the Young’s modulus of PSF films more obviously increased upon adding RSNF, especially at 0.5% loading of RSNF where the increase in modulus was ~40%. Increasing RSNF to higher loadings resulted in decreasing the modulus values, but they were still higher than that of the neat PSF membrane. The improvement in mechanical properties of the films indicates good compatibility between PSF matrix and the surface of RSNF which are rich in lignin with its aromatic ring structures. In addition, formation of hydrogen bonding between hydroxyl groups at the surface of the RSNF and the S=O bonds of PSF could improve mechanical properties of the films. Regarding strain at break, it generally tended to decrease with addition of RSNF; the decrease ranged from 14.5 to 20.7%.

Comparing the improvement in tensile strength properties in the current work to that found in previous work where the so-called “lignin/cellulose nanofibers” complex [30], prepared by sulfuric acid hydrolysis of unbleached pulp and high pressure homogenization in dimethylacetamide, was used with PSF, the maximum improvement in tensile strength was about 50%. Strain at break increased by ~22%, which is in contrast to the strain decrease found in the current work. The tensile modulus was not measured in that work. In another work where cellulose nanofibers from bleached pulp were used with PSF and sulfonated PSF, the maximum improvement in tensile strength and tensile modulus were approximately 20% and 38%, respectively [25]. Comparing the improvement in tensile strength and tensile modulus achieved in the current work to the aforementioned findings clearly shows the advantage of using RSNF isolated from unbleached pulp by direct grinding.

### 3.4. Effect of RSNF on Hydrophilicity and Porosity of PSF Membranes

One of the important reasons for using cellulose in membranes is to increase their hydrophilicity; this affects both water flux across the membrane and its resistance to fouling. The RSNF used still has strong hydrophilicity in spite of the presence of lignin. As shown in Table 4, addition of RSNF to PSF resulted in a decrease in water contact angle even at the smallest RSNF loading where water contact angle was ~90° for the neat PSF membrane and ranged from 80.1° to 82.7° for the different PSF/RSNF samples. The effect of RSNF on water absorption, and thus the calculated porosity, of PSF film was rather more pronounced at 1–2% of RSNF loading. PSF film has poor hydrophilic character and water absorption occurred is mainly due to permeation of water into the porous structure. Increasing porosity as a result of adding RSNF could be due to the higher hydrophilicity, as it is clear from the contact angle values, and wider pores formed at the surface of PSF as a result of presence of RSNF as seen from the SEM images.

### 3.5. Effect of RSNF on Water Flux and Fouling of PSF Films

**Figure 4:**
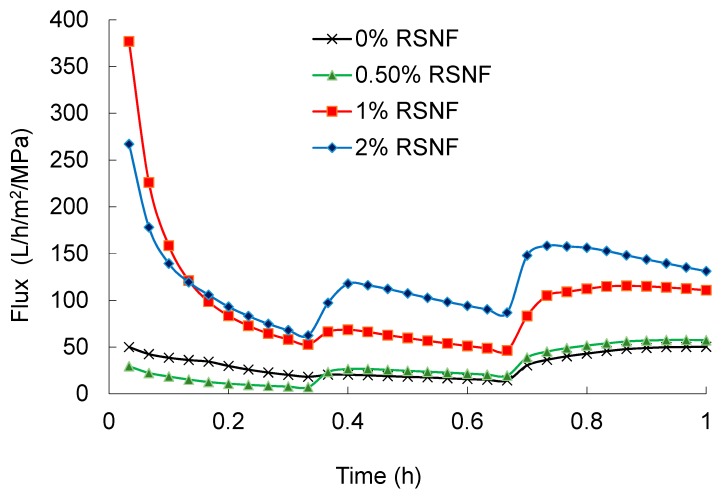
Water flux of PSF and PSF/RSNF membranes with different loadings of RSNF.

**Figure 5:**
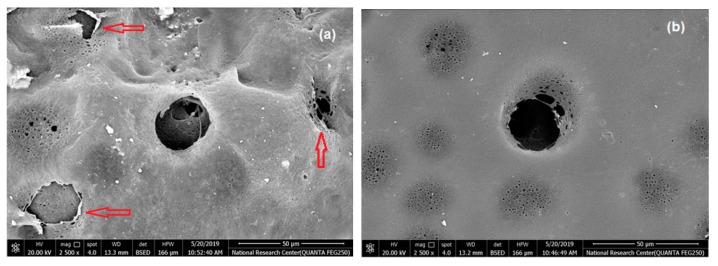
SEM of the PSF/2%RSNF membrane surface: (**a**) after water flux test and (**b**) before water flux test.

Water flux across PSF and PSF/RSNF membranes was tested and the results are presented in Figure 4. Cycles of back pressure were applied every twenty minutes to minimize the decrease of flux as a result of compactness of the film by water pressure. As it is clear in the figure, presence of RSNF with loading 1% and higher had significant effect on water flux. After one hour of the experiment, water flux values of 50 ± 5, 57 ± 5, 111 ± 18, and 131 ± 21 L/h/m^2^/MPa were recorded for neat PSF, PSF/0.5%RSNF, PSF/1%RSNF, and PSF/2%RSNF, respectively. The increase in water flux could be attributed to the higher hydrophilicity, porosity, and water absorption of membrane with higher RSNF contents. The increase in water flux from cycle to another could be due to generation of more pores at the surface by the action of water pressure applied. This is could be seen from the SEM images of membranes’ surface before and after the water flux test (Figure 5). As indicated by the red arrows in the image, the skin layer at the surface was ruptured by the action of water pressure applied generating more pores at the surface.

**Figure 6:**
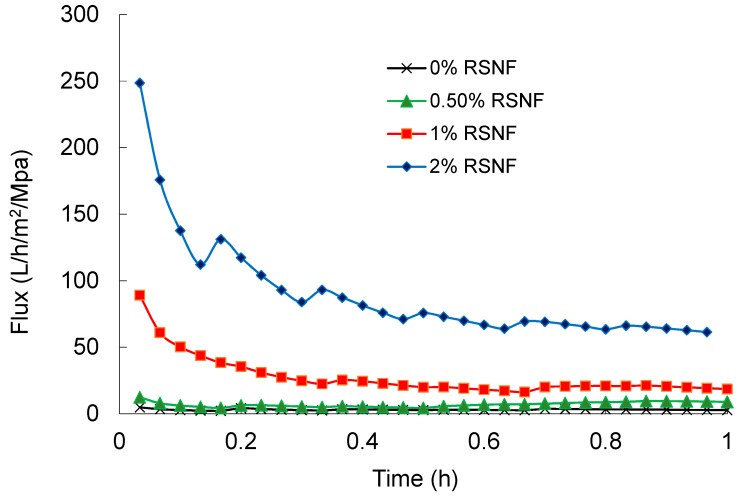
Fouling of PSF and PSF/RSNF membranes with different loadings of RSNF when passing 1% bovine albumin solution.

Regarding fouling of the membranes, as shown in Figure 6, after three cycles of passing protein solution and pure water across the membranes for one hour, water flux values of 2.7 ± 0.26, 8.9 ± 1.75, 18.6 ± 2.48, and 61.3 ± 4.4 L/h/m^2^/MPa were recorded for neat PSF, PSF/0.5%RSNF, PSF/1%RSNF, and PSF/2%RSNF, respectively.

### 3.6. Rejection of Lime Nanoparticles

**Figure 7:**
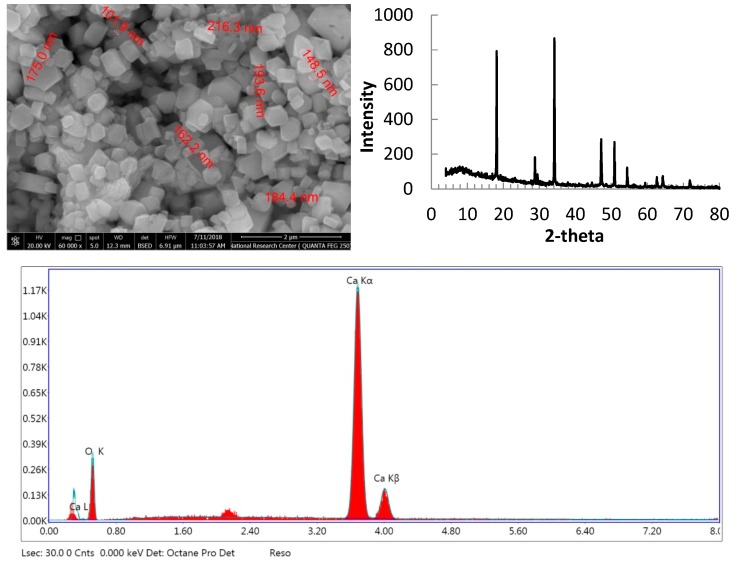
TEM, XRD pattern, and energy-dispersive X-ray spectroscopy (EDS) spectrum of lime nanoparticles.

The prepared membranes were tested for removing lime nanoparticles from dilute suspension containing 2 g/L of lime nanoparticles. The XRD pattern (Figure 7) showed that structure of calcium hydroxide (lime) with hexagonal crystal structure [35]; TEM image showed nanoparticles with diagonal ranged from as low as 100 nm up to several hundred nanometers. The energy-dispersive X-ray spectroscopy (EDS) spectrum proved the purity of the prepared lime nanoparticles. 

The water flux of the lime nanoparticle suspension after 2 h for the different membranes is shown in Table 5. As shown in the table, adding the RSNF resulted in increasing the flux of lime nanoparticles suspension. The maximum increase of water flux was achieved on adding 1–2% of RSNF and was ~850% as compared to that of the neat PSA membrane.

**Figure 8:**
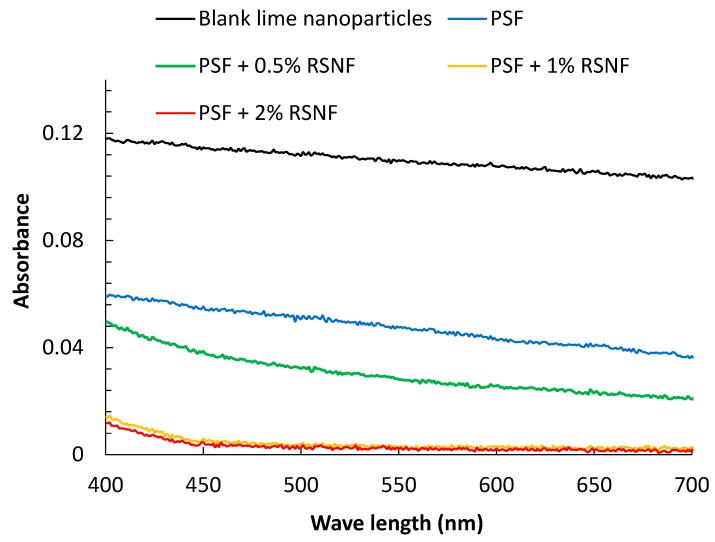
Absorbance spectra of lime nanoparticles suspension and filtrate produced after passing the suspension through the different PSF membranes.

Figure 8 shows visible light absorbance of the filtrate solution after passing through PSF membranes, as well as that of lime nanoparticles suspension with 1 g/L concentration. Interestingly, as shown in the figure, the rejection of lime nanoparticles increased with increasing RSNF content in spite of the increase of diameter of the pores at the surface and porosity of the membranes containing RSNF. The filtrate obtained from filtration using the neat PSF membrane showed a 60% decrease of light absorbance at 600 nm as compared to the blank lime suspension; filtrates obtained from filtration using PSF/0.5%RSNF, PSF/1%RSNF, and PSF/2% RSNF showed 76%, 97%, and 98% decrease in light absorbance, respectively. According to standard curve of lime nanoparticles with different concentrations (not shown), the concentration of lime nanoparticles in the filtrate was 17.4, 11.1, 1.3, and 0.9 mg/L when using PSF, PSF/0.5%RSNF, PSF/1%RSNF, and PSF/2% RSNF membranes, respectively. The increase in rejection of lime nanoparticles with increasing porosity and pore radius at the surface of membranes as the RSNF content increase means that the rejection of lime nanoparticles is not simply by mechanical filtration but involves interaction between the nanoparticles and the functional groups at the RSNF surfaces. RNSF with its very high surface area has plenty of hydroxyl and carboxylic functional groups at their surface which can attract the charged lime nanoparticles. Similar observation was found in previous work in case of using microcrystalline cellulose, cellulose nanocrystals or cellulose nanofibers isolated from bleached pulps when rejection of bovine serum albumin (BSA) was studied [25,27,36]. Although addition of cellulose nanocrystals or nanofibers to PSF resulted in increasing porosity and water flux, rejection of BSA (protein with charged surface) did not significantly affect by the increased porosity or water flux.

## 4. Conclusions

RSNF containing lignin (isolated from unbleached pulp) could be used for improving porosity, hydrophilicity, water flux, and antifouling of PSF membranes at low loadings of RSNF (0.5–2%). Mechanical properties (tensile stress and Young’ modulus) only improved at the lowest RSNF loading (0.5%); higher loadings did not enhance these properties. PSF containing 1–2% of RSNF could be successfully used for removing lime nanoparticles from aqueous suspension at a 10-fold faster rate than in case of using neat PSF, which could only partially remove the lime nanoparticles. The study showed the advantages of using cellulose nanofibers isolated with lower cost than that isolated from bleached pulps for improving properties of PSF related to their use as ultrafiltration membranes.

## Figures and Tables

**Table 1:** Effect of rice straw nanofibers (RSNF) content on viscosity of polysulfone (PSF) solution.

Sample	Viscosity (Pa.s)
PSF	434 ± 0.71
PSF + 0.5% RSNF	533 ± 0.82
PSF + 1.0% RSNF	634 ± 1.41
PSF + 2.0% RSNF	988 ± 5.65

**Table 2:** Diameter of pores at the surface of PSF/RSNF films.

Sample	Diameter of Pores at Surface (µm)
PSF	2.9 ± 1.2
PSF + 0.5% RSNF	5.9 ± 3.4
PSF + 1.0% RSNF	5.8 ± 0.8
PSF + 2.0% RSNF	15.3 ± 8.6

**Table 3:** Tensile strength properties of polysulfone (PSF)/rice straw nanofibers (RSNF) membranes.

Sample	Tensile Strength (MPa)	Tensile Modulus (MPa)	Strain at Maximum Load (%)
PSF	3.88 ± 0.40	164.8 ± 16.2	24.1 ± 2.3
PSF + 0.5% RSNF	5.00 ± 0.51	229.9 ± 27.3	20.6 ± 3.9
PSF + 1.0% RSNF	3.50 ± 0.47	186.9 ± 18.9	19.1 ± 1.7
PSF + 2.0% RSNF	3.84 ± 1.16	176.5 ± 26.6	19.3 ± 2.4

**Table 4:** Water contact angle of the different PSF/RSNF membranes.

Sample	Contact Angle (°)	Water Absorption (%)	Porosity (%)
PSF	89.9 ± 0.4	84.2 ± 2.7	45.5 ± 2.9
PSF + 0.5% RSNF	80.1 ± 5.2	83.6 ± 2.4	54.3 ± 4.6
PSF + 1.0 % RSNF	81.5 ± 4.2	125.7 ± 5.9	69.8 ± 4.8
PSF + 2.0 % RSNF	82.7 ± 1.6	119.6 ± 6.8	66.6 ± 6.1

**Table 5:** Flux of lime nanoparticles suspension through the different membranes after 2 h.

Samples	Flux rate (L/h/m^2^/MPa)
Polysulfone (PSF)	1.027 ± 0.12
PSF/0.5 % RSNF	4.00 ± 0.40
PSF/1 % RSNF	10.27 ± 1.06
PSF/2 % RSNF	10.78 ± 1.67
